# Frailty assessment in older adults using upper-extremity function: index development

**DOI:** 10.1186/s12877-017-0509-1

**Published:** 2017-06-02

**Authors:** Nima Toosizadeh, Christopher Wendel, Chiu-Hsieh Hsu, Edward Zamrini, Jane Mohler

**Affiliations:** 1 Arizona Center on Aging (ACOA), Department of Medicine, University of Arizona, College of Medicine, 1807 E Elm St., PO Box 245072, Tucson, AZ 85724-5072 USA; 2 Division of Geriatrics, General Internal Medicine and Palliative Medicine, Department of Medicine, University of Arizona, Tucson, AZ USA; 3 Department of Biomedical Engineering, University of Arizona, Tucson, AZ USA; 4 Department of Epidemiology and Biostatistics, Mel and Enid Zuckerman College of Public Health, University of Arizona, Tucson, AZ USA; 5 Banner Sun Health Research Institute, Sun City, AZ USA

**Keywords:** Upper-limb movement, Disability, Geriatrics, Wearable sensor, Motor performance

## Abstract

**Background:**

Numerous multidimensional assessment tools have been developed to measure frailty; however, the clinical feasibility of these tools is limited. We previously developed and validated an upper-extremity function (UEF) assessment method that incorporates wearable motion sensors. The purpose of the current study was to: 1) cross-sectionally validate the UEF method in a larger sample in comparison with the Fried index; 2) develop a UEF frailty index to predict frailty categories including non-frail, pre-frail, and frail based on UEF parameters and demographic information, using the Fried index as the gold standard; and 3) develop a UEF continuous score (points scores for each UEF parameter and a total frailty score) based on UEF parameters and demographic information, using the Fried index as the gold standard.

**Methods:**

We performed a cross-sectional validation and index development study within the Banner Medical Center, Tucson, and Banner Sun Health Research Institute, Sun City, Arizona. Community-dwelling and outpatient older adults (≥60 years; *n* = 352; 132 non-frail, 175 pre-frail, and 45 frail based on Fried criteria) were recruited. For the UEF test, each participant performed a 20-s elbow flexion, within which they repetitively and rapidly flexed and extended their dominant elbow. Using elbow motion outcomes two UEF indexes were developed (categorical and score). The Fried index was measured as the gold standard.

**Results:**

For the categorical index, speed of elbow flexion, elbow range of motion, elbow moment, number of flexion, speed variability and reduction within 20 s, as well as body mass index (BMI) were included as the pre-frailty/frailty predictor parameters. Results from 10-fold cross-validation showed receiver operator characteristic area under the curve of 0.77 ± 0.07 and 0.80 ± 0.12 for predicting Fried pre-frailty and frailty, respectively. UEF score (0.1 to 1.0) was developed using similar UEF parameters.

**Conclusions:**

We present an objective, sensor-based frailty assessment tool based on physical frailty features including slowness, weakness, exhaustion (muscle fatigue), and flexibility of upper-extremity movements. Within the current study, the method was validated cross-sectionally using the Fried index as the gold standard and the UEF categorical index and UEF frailty score were developed for research purposes and potentially for future clinical use.

**Electronic supplementary material:**

The online version of this article (doi:10.1186/s12877-017-0509-1) contains supplementary material, which is available to authorized users.

## Background

Older adult healthcare decisions can be complicated due to the heterogeneity of the aging population [1, 2]. There is increasing interest in risk stratification in older adult to direct individualized management strategies, in support of the triple aim. Frailty syndrome, defined as “a state of increased vulnerability to poor resolution of homeostasis after a stressor event as a consequence of accumulated age-related defects in physiological systems”, is increasingly turned to as a risk stratification strategy [3]. Frailty affects approximately 11% of community-dwelling older adults and as many as 30–70% of older surgical patients [4, 5], and has been associated with adverse health-related outcomes including increased complications, impaired balance and fall, hospitalization, length of hospital stay, and mortality [6–9].

Although numerous multidimensional assessment tools have been developed to measure frailty, there are many feasibility issues related to these methods, and there is no universal “clinical standard” for assessment of frailty [10–12]. The Fried Frailty index and the Rockwood score, as the most commonly used frailty measures, have been validated within large samples [6, 7]. However, practical issues often limit the application of these approaches in clinical settings. These issues include the space, safety, and ability requirements for gait assessment for the Fried index, the need for multiple pieces of clinical information for the Rockwood score, and the time required for assessment for both measures [12].

We previously developed an upper-extremity function (UEF) assessment method, showing convergent validity to the Fried index and the Rockwood score [13, 14], and high correlation with the 6-min walk test [15]. The UEF assessment method is objective and quick and integrates low-cost sensors, the physical task is performed in less than 1 min, and the post-processing takes less than 2 min (see Additional file 1: Text S1 and Additional file 2: Text S2). The uniqueness of the proposed approach is its applicability for both ambulatory and non-ambulatory elders. Since we previously demonstrated association between UEF and frailty, the purpose of the current study was to: 1) cross-sectionally validate the UEF method in a larger sample in comparison with the Fried index; 2) develop a UEF frailty index to predict frailty categories including non-frail, pre-frail, and frail based on UEF parameters and demographic information, using the Fried index as the gold standard; and 3) develop a UEF continuous score (points scores for each UEF parameter and a total frailty score) based on UEF parameters and demographic information, using the Fried index as the gold standard.

## Methods

### Participants

Community-dwelling and outpatient older adults (≥60 years) were recruited from Banner University Medical Center in Tucson and from the Banner Sun Health Research Institute in Sun City, Arizona. Exclusion criteria included: known disorders associated with severe motor deficits (including stroke, diagnosed Parkinson’s disease, and amputation); major mobility disorders (e.g., who were unable to walk a distance of ~10 m); and upper-extremity disorders (including severe bilateral shoulder or elbow rheumatoid or osteoarthritis). Assistive devices used routinely by participants in their daily activities, including canes and walkers, but excluding wheelchairs, were allowed. The study was approved by the University of Arizona and Banner Sun Health Research Institute’s Institutional Review Boards. Before participation, written informed consent according to the principles expressed in the Declaration of Helsinki [16] was obtained from all subjects (or an authorized person in case of lack of clinically assessed capacity for informed consent).

### Frailty and cognitive evaluation

The Fried index was used as the gold standard, based on unintentional weight loss, self-reported exhaustion and low physical activity, weakness (grip strength), and slow walking speed [6]. Grip strength was assessed three times for right and left hands, and the average for both sides was reported. Walking speed was measured as the required time to walk 4.6 m (15 ft). Individuals were classified as frail with three or more positive Fried criteria, as pre-frail with one or two criteria, and as non-frail with none. In addition, mini-mental state examination (MMSE) [17], Montreal cognitive assessment (MoCA) [18], or both were collected to assess cognitive condition.

### UEF procedure and outcomes

Per our previously validated UEF method [14], wearable motion sensors (tri-axial wearable gyroscope sensor, BioSensics LLC, Cambridge, MA) were used to measure forearm and upper-arm motion, and ultimately the elbow angular velocity. Each participant performed a 20-s trial of elbow flexion, within which they repetitively and fully flexed and extended their dominant elbow as quickly as possible in the seated position, while wearing the UEF system. Before the actual test, participants performed a short practice trial to become familiar with the protocol. The protocol was explained to participants and they were encouraged only once, before elbow flexion, to do the task as fast as possible (participants were not further encouraged during the task). All assessments were performed by trained researchers, and to assure consistency, they provided exactly the same verbal instruction before measurement: “As quickly as you can, bend and straighten your dominant arm over and over for 20 s. Make sure you straighten and bend your arm all the way”.

Several outcome measures representing kinematics and kinetics of elbow flexion were derived to quantify “slowness”, “weakness”, “exhaustion”, and “flexibility” as frailty markers [6, 19]. Outcome measures included: 1) speed; 2) flexibility; 3) power; 4) rise time; 5) moment; 6) speed variability; 7) speed reduction; and 8) flexion number (see Table 1 for definitions) [14]. Readers are referred to previous work [13, 14] for more details regarding validation of UEF and parameter descriptions.

**Table 1:** UEF parameter definitions

Parameter	Definition
Speed	Mean value of the elbow angular velocity range (maximum minus minimum speed)
Flexibility	Mean value of the elbow flexion range
Power	Mean value of the product of the angular acceleration range and the range of angular velocity
Rise time	Mean value of the time required to reach the maximum angular velocity
Moment	Mean value of the maximum moment on elbow within each flexion/extension; estimated from the moment of inertia of the forearm and the hand, and elbow motion
Speed variability	Coefficient of variation (standard deviation divided by the mean) of the angular velocity range
Speed reduction	Difference in the angular velocity range between the last and the first 5 s of elbow flexion as a percentage of the initial angular velocity range
Flexion number	Number of flexion/extensions during 20 s

### Statistical analysis and UEF index development

Sociodemographic parameters were compared between three Fried frailty groups using separate analyses of variance (ANOVAs) or chi-square (χ^2^). UEF parameters were compared between males and females and correlations between UEF parameters and body mass index (BMI) were determined.

Two UEF indexes were developed. The first index (UEF categorical index) was developed to provide frailty status as non-frail, pre-frail, and frail, similar to the Fried index. Multivariable ordinal logistic models with the Fried frailty categories as the dependent variable, and UEF parameters plus demographic information (i.e., age, sex, and BMI) as independent variables were used to develop the UEF categorical index. To construct models, the following steps were followed: 1) descriptive analysis of UEF parameters: outliers detection within each frailty group using box plots and histograms and testing of distribution normality using Shapiro-Wilk *W* test; 2) univariate analysis for UEF parameters as independent variables: UEF parameters with significant association with the Fried frailty status were selected for subsequent steps; 3) testing of collinearity between UEF parameters: using variance inflation factor (VIF) values. A VIF cutoff value larger than 10 was considered an indication of presence of collinearity [20]; 4) stepwise parameter selection: UEF and demographic parameters were selected based on Akaike information criterion (AIC) values. Since BMI has been shown to have a U-shape association with frailty [21], both continuous and categorical variables (<20, 20–24.9, 25–29.9, 30–34.9, ≥ 35 kg/m^2^) were tested in separate analyses; and 5) model evaluation: using 10-fold cross-validation, in which the sample was randomly divided into k = 10 equal parts. At each k^th^ iteration, k − 1 partitions were used as the training dataset and the left out partition was used as the validation dataset. The average values of area under receiver operating characteristic (ROC) curves, AIC, and accuracy – (true positive + true negative) / sample size – were calculated.

Parameters selected from the categorical index were used to develop the UEF continuous score, using methods developed for the Framingham cardiovascular risk score [22]. For developing the UEF score, first, each continuous UEF parameter and BMI were split into three categories based on mean values for each frailty group. That is parameter mean values were considered as reference values (W) and the mid-points between reference values were considered as cut-offs. Next, the distance between each category and the lowest (reference) category in regression units was determined, by multiplying the β (parameter estimates from the categorical index model) by the difference between the category W and the reference category W_REF_, or β (W-W_ref_). Then, each categorical independent variable was assigned a point value by dividing the distance β (W-W_ref_) by a base constant that represents a coefficient value corresponding to one point. Point values were rounded to the nearest integer. For simplicity, age (increased risk of frailty associated with a 4-year increase in age corresponding to age differences between frailty groups) was selected as the base constant, as it provided the smallest distance β(W-W_ref_). Readers are referred to Additional file 2: Text S2 for details regarding UEF scorings. The UEF score (0 extreme resilience and 1 extreme frailty) for a given participant was defined as the sum of points corresponding to performance results and demographic information.

The UEF score distribution was plotted. Based on previous reports on frailty index/score distribution [23, 24], the UEF score was expected to show a skewed gamma distribution. Lastly, associations between the UEF score with age, the Fried index, and MMSE and MoCA scores were investigated using both Pearson and Spearman Rank correlations.

## Results

### Participants

Three hundred fifty-two participants were recruited including 132 (37%) non-frail, 175 (50%) pre-frail, and 45 (13%) frail older adults, defined by the Fried index. Age, height, and MMSE score were significantly different among frailty groups (*p* < 0.01). On average, frail participants were ~3 years older and 1% shorter compared to pre-frail individuals; pre-frail participants were ~4 years older and 2% shorter compared to non-frails. Non-frail participants had 2% higher MMSE score compared to other groups. Sociodemographic parameters are presented in Table 2. Significant differences were observed in power, flexibility, and moment UEF parameters between male and female participants (*p* < 0.03). Male participants had 32% and 99% larger elbow power and moment, respectively, and were 3% less flexible in elbow motion compared to females. Also, participants with higher BMI showed less power (*r* = −0.13, *p* < 0.01) and higher elbow moment (*r* = 0.17, *p* < 0.01).

**Table 2:** Participants’ sociodemographic characteristics, Fried criteria, and UEF parameters for three frailty categories defined using the Fried index

Variable	Non-frail	Pre-frail	Frail	*p*-value (ES)
Number, n (% of total)	132 (37%)	175 (50%)	45 (13%)	-
Male, n (% of the group)	52 (39%)	59 (34%)	12 (27%)	0.26
Age, year (SD)	75.95 (7.45)	80.20 (9.70)	83.62 (7.09)	<0.001* (0.33)
Height, cm (SD)	165.91 (11.40)	163.20 (9.40)	161.32 (9.48)	0.01* (0.16)
Weight, kg (SD)	74.24 (17.20)	73.76 (18.56)	74.26 (17.95)	0.97 (0.01)
Body mass index, kg/m^2^ (SD)	26.79 (4.61)	27.60 (6.21)	28.56 (6.56)	0.17 (0.10)
Mini-mental state examination, 0–30 (SD) (Data from 286 participants)	28.92 (1.23)	28.27 (1.90)	28.28 (1.86)	<0.01* (0.02)
Montreal cognitive assessment, 0–30 (SD) (Data from 134 participants)	25.26 (3.44)	23.96 (4.01)	23.30 (3.61)	0.09 (0.20)

The asterisk symbol represent a significant difference between frailty groups. Only comparisons of UEF parameters were adjusted for age, sex, and body mass index (BMI)

### UEF categorical index

Based on graph inspections and Shapiro-Wilk tests, UEF parameter distributions appeared normal, with exception of power and moment, and these two exceptions appeared normally distributed after logarithmic transformation. Univariate logistic models revealed that all UEF parameters were significantly associated with the Fried frailty status (*p* < 0.001). VIF testing demonstrated collinearity of speed and power, when both were present in the model; removing either of these two parameters removed the indication of collinearity for the other. Both parameters showed equal univariate effect size and produced similar multivariable model fit. We selected speed of elbow flexion as a predictor parameter for the model and excluded power from further analyses.

Using UEF parameters, (except power) in the forward stepwise approach, resulted in speed, flexibility, moment, speed variability, speed reduction, and flexion number as included parameters (Table 3). Of note, when age, sex, and BMI were individually entered into the logistic model in addition to UEF parameters only continuous BMI was significantly associated with the Fried index. Therefore, we removed age and sex as predictor parameters since frailty and pre-frailty predictions (ROC area under curve and accuracy) remained unchanged after removing these parameters. Using the ordinal logistic regression model, the final probability equation was derived from parameter estimates (Additional file 1: Text S1). Using the model with the whole sample, ROC area under curves were 0.86 and 0.91 for predicting pre-frail and frailty, respectively; respective values of AIC and accuracy were 461.72 and 0.70. Results from 10-fold cross-validation showed mean (standard deviation - SD) values of 0.77 (0.07), 0.80 (0.12), 56.73 (5.17), and 0.69 (0.08) for ROC area under curve for pre-frailty, ROC area under curve for frailty, AIC, and accuracy, respectively.

**Table 3:** UEF categorical index: results of the multivariable ordinal logistic model

Independent variables	Parameter estimates	Standard errors	chi-square (χ2)	*p*-value	95% CI (Lower)	95% CI (Upper)
Intercept, [non-frail]	−2.6304	2.0702	1.61	0.20	−6.5822	1.5641
Intercept, [pre-frail]	1.5140	2.0759	0.53	0.47	−2.4535	5.7244
Speed, deg./s	0.0025	0.0010	6.12	0.01*	0.0005	0.0045
Flexibility, deg	0.0207	0.0069	8.90	<0.01*	0.0073	0.0345
Log (Moment), Nm	0.7176	0.3278	4.79	0.03*	0.0870	1.3627
Speed variability, %	−0.0441	0.0206	4.55	0.03*	−0.0847	−0.0058
Speed reduction, %	−0.0342	0.0153	4.98	0.03*	−0.0647	−0.0045
Flexion number, n	0.0647	0.0284	5.96	0.01*	0.0099	0.1205
Sex, [female]	0.1214	0.1570	0.60	0.44	−0.1894	0.4341
Age, year	−0.0206	0.0158	1.70	0.19	−0.0514	0.0099
BMI, kg/m^2^	−0.0611	0.0284	5.96	0.01*	−0.1112	−0.0126

Dependent variable: Fried frailty categories; independent variables: UEF parameters, age, sex, and body mass index (BMI). The asterisk symbol represents a significant independent association

### UEF score

Highest UEF sub-scores were obtained for speed and flexibility UEF parameters (Additional file 2: Text S2). Similar to the UEF index, age and sex were removed as they demonstrated non-significant association with frailty. Scores with equivalent categorical cutoffs are presented in Additional file 2: Text S2. As expected, the UEF score histogram was described by a gamma distribution (*p* < 0.01). Results from Pearson tests showed significant positive correlations between the UEF score with MMSE (*r* = 0.22, *p* < 0.001) and MoCA (*r* = 0.37, *p* < 0.001), and significant negative correlation between the UEF score and age (*r* = −0.37, *p* < 0.001). Spearman Rank correlations between the same pairs of variables showed no appreciable difference from the Pearson correlations.

## Discussion

### UEF and frailty

As hypothesized, UEF parameters were significantly associated with frailty and UEF accurately predicts Fried frailty categories. The theorized biological mechanism is that sarcopenia/dynapenia, as a manifestation of physical frailty, would influence the entire muscular structure of the human body, including both lower and upper-extremities [6, 25]. Therefore, speed of upper-extremity movement was measured as a surrogate of gait speed. Associations between frailty and weakness, exhaustion, and flexibility have also been investigated in several studies. Grip strength weakness measure and exhaustion doing physical activity demonstrated strongest association with adverse health outcomes [6, 26]. Of note, rather than exhaustion within daily physical activity, proposed UEF speed variation and reduction parameters during maximum pace elbow flexion assess muscle fatigue, which is defined as inability to maintain required/maximum force generating capacity of the muscle [27]. Assessing muscle fatigue for screening physical frailty has been recommended previously, because frailty and muscle fatigue share several contributory factors aside from aging, including inflammation, physical inactivity, malnutrition, hormonal deficiencies, as well as muscular factors such as muscle strength and size [28, 29]. Finally in regard to the UEF flexibility parameter, although limited research exists, Brown et al. reported significantly less range of motion in the shoulder joint among older adults with greater frailty [19].

As mentioned above, all UEF parameters were selected based on frailty features. Although direct association of a parameter with frailty is necessary, it is not sufficient for including it into a frailty index. There are five criteria that have been recently proposed by Searle et al., as requirements for frailty index parameters: 1) parameters must associate with health status; 2) parameters should increase in prevalence with age; 3) parameters should not saturate at early age; 4) selected parameters as a group must cover a wide range of frailty features; and 5) frailty index must contain the same parameters for different measurements on the same sample [24]. UEF parameters and index cover all of these five criteria. Each parameter is associated with age and health status, and would not saturate at early ages as it incorporate maximum pace muscle performance (criteria 1, 2 and 3). Further, the UEF index covers several features of frailty, and it incorporates a single algorithm based on sensor-based parameters (criteria 4 and 5).

### UEF and participants’ characteristics

Among demographic data, only BMI was a significant frailty predictor; age and sex were not independently associated with frailty (Table 3). Current findings suggest that older adults with higher BMI (> 27 kg/m^2^) were at higher risk of frailty. Reports from previous work revealed that there is a U-shape association between BMI and frailty; the level of frailty was higher at very low BMI (20 kg/m^2^) indicating wasting, and very high (30 kg/m^2^) BMI indicating obesogenic sarcopenia [21]. We did not observe an association between very low BMI and greater frailty, probably due to our small sample size and the fact that components of the implemented gold standard (i.e., the Fried index) has already been adjusted for BMI.

As presented above, UEF flexibility and moment parameters were different between sexes. It was expected that male participants would show higher strength in performing elbow flexion test as reflected within the moment UEF parameter. On the other hand, previous research suggested that older females have larger maximum voluntary range of motion of elbow and shoulder joints when compared to males [30]. We believe sex was not influential in the UEF model since sex-differences in UEF parameters cancel out the overall influence of sex in the model.

### Limitations and future direction

First, the current study lacked test-retest assessments. The assessment procedure is objective and requires minimum judgement from the test provider, and, therefore, we believe UEF would provide high test-retest reliability, as it was also evident from cross-validation results. Second, the UEF index and score application needs to be confirmed in prospective longitudinal studies for predicting health outcomes. Especially, the UEF index/score validity and reliability for predicting mortality, hospitalization, exacerbation, as well as in-hospital outcomes such as length of stay, discharge disposition, and readmission should be investigated in future research. Within the current study, UEF index and score were developed cross-sectionally; we validated the UEF accuracy for predicting frailty using the Fried index as the “gold standard”. However, the Fried index includes only physical measures and lacks other components of frailty such as cognitive impairment, depression, and comorbidity. Third, the focus of the UEF test was solely on physical frailty and lacks a measure of cognition. Although we showed associations between UEF score and cognitive tests (i.e., MMSE and MoCA), no direct conclusion can be made regarding cognitive frailty. As a future direction, UEF using dual-task performance may be a sensitive marker of cognitive status, based on simultaneous assessment of physical frailty and cognitive performance [31].

## Conclusions

We present a novel sensor-based frailty assessment tool. Findings demonstrated acceptable accuracy (~0.70) in predicting frailty status in comparison with the Fried index as the gold standard. The assessment was based on physical frailty features including slowness (speed of elbow flexion), weakness (strength of upper-extremity muscles), exhaustion (muscle fatigue), and flexibility (upper-extremity range of motion). The UEF assessment method is objective and integrates low-cost reusable sensors (available for as low as $200). The physical assessment (including preparation/calibration) is easily performed in less than 1 min, and post-processing to obtain the categorical and continuous score is performed in less than 2 min. The test is practical for busy clinical settings and alleviates fall risk. The scoring (data analysis) process can be performed by a computer or cellphone using a free web-based software (uef.aging.arizona.edu).

## Additional file 1

Additional file 1: Text S1. Categorical upper-extremity function index. (DOCX 15 kb)
Additional file 2: Text S2. Upper-extremity function score. (DOCX 16 kb)

## Abbreviations

AIC: Akaike information criterion
ANOVA: Analysis of variance
BMI: Body mass index
MMSE: Mini-mental state examination
MoCA: Montreal cognitive assessment
ROC: Receiver operating characteristic
UEF: Upper-extremity function
VIF: Variance inflation factor
