# Supplementary material for: Frailty assessment in older adults using upper-extremity function: index development
Source: BMC Geriatr. 2017 Jun 2;17:117. doi: 10.1186/s12877-017-0509-1 (PMC5457588; doi:10.1186/s12877-017-0509-1)
Supplement: Additional file 1: Text S1. — Categorical upper-extremity function index. (DOCX 15 kb) [file 12877_2017_509_MOESM1_ESM.docx]

**Additional file 1: Text S1**

UEF categorical index: The probability equation can be derived using parameter estimates ($\beta_{i}$) and magnitude of independent variables ($a_{i}$) as follows (see Supplementary Table S1 for $\beta_{i}$ values and the list of independent variables):

$P\left[ non-frail \right]=\frac{1}{1+exp-(\beta_{1}+\beta_{3}a_{3}+\beta_{4}a_{4}+\ldots+\beta_{9}a_{9})}$ (A1)

$P\left[ pre-frail \right]=\frac{1}{1+exp-(\beta_{2}+\beta_{3}a_{3}+\beta_{4}a_{4}+\ldots+\beta_{9}a_{9})}-\frac{1}{1+exp-(\beta_{1}+\beta_{3}a_{3}+\beta_{4}a_{4}+\ldots+\beta_{9}a_{9})}$ (A2)

$P\left[ frail \right]=1-\frac{1}{1+exp-(\beta_{2}+\beta_{3}a_{3}+\beta_{4}a_{4}+\ldots+\beta_{9}a_{9})}$ (A3)

| **Independent Variables** | **Parameter Estimates** |
| --- | --- |
| Intercept, [non-frail] | β_1_ = -5.0064 |
| Intercept, [pre-frail] | β_2_ = -0.9009 |
| $a_{3}$: Speed, deg/s | β_3_ = 0.0027 |
| $a_{4}$: Flexibility, deg | β_4_ = 0.0221 |
| $a_{5}$: Log (Moment), Nm | β_5_ = 0.6202 |
| $a_{6}$: Speed variability, % | β_6_ = -0.0406 |
| $a_{7}$: Speed reduction, % | β_7_ = -0.0345 |
| $a_{8}$: Flexion number, n | β_8_ = 0.0647 |
| $a_{9}$: BMI, kg/m^2^ | β_9_ = -0.0668 |

Table S1: UEF categorical index, Independent variables ($a_{i}$) and parameter estimates ($\beta_{i}$) are presented

A free web-based UEF index calculator available to research collaborators at [uef.aging.arizona.edu](file:///C:\Neema\WORK\Index%20Paper\uef.aging.arizona.edu).
