# Supplementary material for: Frailty assessment in older adults using upper-extremity function: index development
Source: BMC Geriatr. 2017 Jun 2;17:117. doi: 10.1186/s12877-017-0509-1 (PMC5457588; doi:10.1186/s12877-017-0509-1)
Supplement: Additional file 2: Text S2. — Upper-extremity function score. (DOCX 16 kb) [file 12877_2017_509_MOESM2_ESM.docx]

**Additional file 2: Text S2**

Table S2: UEF score: The total score = (54 - total point) / 54

0: Extreme resilience level

1: Extreme frailty level

Frailty cutoffs: < 0.3: non-frail; 0.3 – 0.6: pre-frail; and > 0.6 frail (Cutoffs were developed based on mean UEF scores within each Fried frailty group)

| **Scoring** | | | **Score Calculation** | | | |
| --- | --- | --- | --- | --- | --- | --- |
| **Variable** | **Variable Ranges** | **Points (0-54)** | **Parameter Estimate (β)** | **Reference Value (W)** | **β (W-W_ref_)** | **Points**  **β (W-W_ref_) / B_ref_ †** |
| Speed, deg/s | < 670 | 0 | 0.0027 | 535 | 0 | 0 |
|  | 670 - 950 | 9 |  | 812 | 0.75 | 9.08 |
|  | > 950 | 18 |  | 1099 | 1.52 | 18.48 |
| Flexibility, deg | < 95 | 0 | 0.0221 | 84 | 0 | 0 |
|  | 95 - 120 | 6 |  | 107 | 0.51 | 6.17 |
|  | > 120 | 11 |  | 125 | 0.91 | 10.99 |
| Moment, Nm | < 0.6 | 0 | 0.6202 | -0.41 | 0 | 0 |
|  | 0.6 - 1 | 2 |  | -0.1 | 0.19 | 2.33 |
|  | > 1 | 4 |  | 0.08 | 0.30 | 3.69 |
| Speed variability, % | < 10 | 7 | -0.0406 | 9 | 0 | 0 |
|  | 10-17 | 5 |  | 12 | -0.12 | -1.48 |
|  | > 17 | 0 |  | 22 | -0.53 | -6.41 |
| Speed reduction, % | < 5 | 5 | -0.0345 | 3 | 0 | 0 |
|  | 5 - 10 | 4 |  | 6 | -0.10 | -1.26 |
|  | > 10 | 0 |  | 15 | -0.41 | -5.02 |
| Flexion number, n | < 18 | 0 | 0.0647 | 15 | 0 | 0 |
|  | 18 - 23 | 5 |  | 21 | 0.39 | 4.71 |
|  | > 23 | 8 |  | 25 | 0.65 | 7.85 |
| BMI, kg/m^2^ | ≤ 27 | 1 | -0.0668 | 26.8 | 0 | 0 |
|  | > 27 | 0 |  | 27.6 | -0.05 | -0.65 |

† B_ref_ (base constant): age parameter estimate x average age differences between frailty groups = 0.0206 x 4

A free web-based UEF scoring system available to research collaborators at [uef.aging.arizona.edu](file:///C:\Neema\WORK\Index%20Paper\uef.aging.arizona.edu).
